# Penile cancer and lymph node management: a call for standardization

**DOI:** 10.1007/s11255-025-04911-6

**Published:** 2025-11-19

**Authors:** Achille Aveta, Vincenzo Iossa, Gianluca Spena, Roberto Contieri, Alessandro Izzo, Francesco Passaro, Antonio Tufano, Vittorio Imperatore, Sisto Perdonà, Lorenzo Romano, Carlo Giulioni, Angelo Cafarelli, Pierluigi Russo, Fabio Crocerossa, Francesco Lasorsa, Giuseppe Lucarelli, Matteo Ferro, Raffaele Balsamo, Celeste Manfredi, Davide Arcaniolo, Salvatore Siracusano, Savio Domenico Pandolfo

**Affiliations:** 1 Department of Urology, Istituto Nazionale Tumori IRCCS, “Fondazione G. Pascale”, Naples, Italy; 2 Department of Urology, Azienda Ospedaliera “S.G. Moscati”, Avellino, Italy; 3 Department of Urology, San Carlo di Nancy Hospital, Rome, Italy; 4 Department of Woman, Child and General and Specialized Surgery, Università degli Studi della Campania “Luigi Vanvitelli”, 81100 Naples, Italy; 5 Urology Unit, Villa Igea Private Hospital, Ancona, Italy; 6 Department of Urology, IRCCS A. Gemelli University Polyclinic Foundation, Rome, Italy; 7 Department of Life Science, Health, and Health Professions, IRCCS A. Gemelli University Polyclinic Foundation, Rome, Italy; 8 Department of Urology, Magna Graecia University of Catanzaro, 88100 Catanzaro, Italy; 9 Urology and Kidney Transplantation Unit, Department of Precision and Regenerative Medicine and Ionian Area-Urology, University of Bari “Aldo Moro”, 70124 Bari, Italy; 10 Urology Unit, IRCCS Istituto Tumori “Giovanni Paolo II”, 70124 Bari, Italy; 11 Unit of Urology, Department of Health Science, University of Milan, ASST Santi Paolo and Carlo, Via A. Di Rudini 8, 20142 Milan, Italy; 12 Urology Unit, AORN Ospedali dei Colli, Monaldi Hospital, 80131 Naples, Italy; 13 Department of Urology, University of L’Aquila, L’Aquila, Italy; 14 Department of Neurosciences, Science of Reproduction and Odontostomatology, Federico II University, Naples, Italy

**Keywords:** Penile cancer, Lymph node management, Inguinal lymph node dissection, penile cancer guidelines

## Abstract

**Purpose:**

This study aims to compare and organize recommendations from the most eminent international guidelines on the staging and treatment of lymph node (LN) involvement in penile cancer (PC). Early recognition and appropriate management of nodal disease remain the cornerstone of care, influencing both survival and treatment-related morbidity.

**Methods:**

This study compares and organize recommendations form the most eminent international guidelines—including NCCN, ESMO-EURACAN, and EAU-ASCO—on the staging and treatment of lymph node involvement in penile cancer.

**Results:**

Open inguinal LN dissection (ILND) remains the standard treatment for not superficial cancers and its morbidity has driven interest in minimally invasive surgical approaches, such as video-endoscopic inguinal lymphadenectomy (VEIL), including its robot-assisted variant (RA-VEIL). Their role is well defined in clinically node-negative (cN0) patients but remains investigational in node-positive (cN+) cases. For cN3 disease and pelvic node involvement, multimodal strategies including chemotherapy, surgery, and radiotherapy are required.

**Conclusion:**

The variability among guidelines underscores the need for collaborative efforts and high-quality prospective trials to refine and standardize treatment recommendations.

## Introduction

Penile cancer (PC) is a rare malignancy with significant geographic variation in its incidence. In the most high-income countries, it affects fewer than 1 in 100,000 men annually, whereas in certain low- and middle-income regions, particularly parts of Africa, Asia, and South America, it may account for up to 10% of all male cancers. This disparity is largely attributed to differences in hygiene, circumcision practices, HPV infection prevalence, and access to early diagnosis and treatment [1].

Regional lymph node (LN) management remains a cornerstone of surgical management. Indeed, one of the key prognostic factors for survival is the presence of LN metastases, with 5-year cancer-specific survival rates varying between 20 and 80%, depending on the node involvement or N stage [2, 3]. Consequently, timely and accurate LN assessment and dissection are essential.

Due to the high morbidity historically associated with open radical inguinal LN dissection (rILND), with complications reported in up to 61% of cases, there has been a growing interest in minimally invasive techniques aimed at reducing surgical burden [4]. Open inguinal LN dissection (ILND) remains the gold standard treatment; however, its significant complication profile, including wound infections (2–43%), skin necrosis (3–50%), lymphoedema (3.1–30%), lymphocele formation (1.8–26%), and seroma (2.4–60%), has prompted the development of alternative approaches such as video-endoscopic inguinal lymphadenectomy (VEIL) and its robot-assisted variant (RA-VEIL) [5–7].

Despite technological progresses, nodal management is still an area of clinical uncertainty. Variability in treatment recommendations reflects the scarcity of high-level evidence, particularly for in case of nodal involvement and metastatic disease, leading to inconsistent adherence to guidelines in clinical practice [8]. For instance, a recent analysis reported a 26.3% rate of non-adherence to European Association of Urology (EAU) guidelines in lymph node (LN) management [9].

Although recent systematic reviews have summarized the available evidence, they do not examine how major societies translate this evidence into recommendations [10]. Our objective is, therefore, to compare the main international guidelines, highlighting areas of alignment and disagreement that influence real-world LN management drawing from the most recent versions issued by European Association of Urology (EAU)-American Society of Clinical Oncology (ASCO), European Society for Medical Oncology-European Reference Network on Rare Adult Solid Cancers (ESMO-EURACAN), and National Comprehensive Cancer Network (NCCN). Special emphasis is placed on controversial areas such as the role of minimally invasive lymphadenectomy, indications for pelvic node dissection, and evolving strategies for systemic therapy in advanced disease [5, 11, 12].

## Evidence synthesis

A summary of the methodological aspects and recommendations of the EAU-ASCO, ESMO-EURACAN, and NCCN guidelines on PC management is outlined in Table 1. All guidelines incorporate systematic summaries of evidence, albeit with variations in methodology and scope. EAU-ASCO are based on structured systematic reviews of the literature, integrating data from Medline, EMBASE, and Cochrane Libraries, with ongoing systematic reviews expected to refine future updates. ESMO-EURACAN follows a consensus-driven approach, incorporating available literature but emphasizing expert opinion where evidence is limited. NCCN guidelines, in contrast, prioritize expert consensus and panel discussions while recommending clinical trial participation whenever possible. EAU-ASCO explicitly reports Level of Evidence (LE) and Strength of Evidence (SE), ESMO-EURACAN evaluates its recommendations using a grading system based on levels of evidence (I–III) and strength of recommendations (A–C), while NCCN rely more on expert consensus without always specifying them. These different approaches contribute to differences across international guidelines. Moreover, while some indications have specific recommendations, while others lack formal guidance due to limited evidence.

**Table 1:** Summary of methodological aspects of the EAU-ASCO, ESMO-EURACAN, and NCCN guidelines

	ESMO-EURECAN	NCCN	EAU-ASCO
Year of publication	2024	2025	2024
Systematic review of evidence	Expert-selected literature, no systematic review	Not systematic, consensus-based	Systematic reviews conducted for selected topics
Evaluation of quality	Incomplete GRADE, level-based ranking (i.e., not all questions)	Consensus-based grading system	Incomplete application of GRADE (i.e., not all questions)
Reported link from evidence to recommendation	LoE-based grading, expert consensus. no formal benefit-risk analysis	No direct evidence-to-recommendation link	Balance between benefits and risks of management strategies, evidence quality (including certainty of estimates)
Level of evidence (LE)	I (high) to III (low)	Not consistently specified, often inferred from expert consensus	LE 1 (high) to LE 4 (low)
Strength of evidence (SE)	Grade A (strong), B (moderate), and C (weak)	Rarely explicitly stated, primarily relies on expert opinion	Strong, moderate, weak
Includes patient values and preferences	No formal patient involvement	Patient advocate included—unclear impact	Patient advocate included—unclear impact

*EAU-ASCO* European Association of Urology-American Society of Clinical Oncology, *ESMO-EURACAN* European Society for Medical Oncology-European Reference Network on Rare Adult Solid Cancers, *NCCN* National Comprehensive Cancer Network, *LN* lymph node, *PC* penile cancer, *LoE* level of evidence, *GRADE* Grading of Recommendations, Assessment, Development, and Evaluations

## Results

### Clinically node-negative disease (cN0)

Since clinical LN TNM staging relies on physical examination to evaluate LN involvement, cN0 is defined by the absence of palpable inguinal LN. However, this approach, especially in obese patients, can miss micrometastases in up to 25% of cases [13]. As non-invasive methods (nomograms/imaging) are inadequate for detecting micrometastatic disease, surgical staging remains essential. Yet only 20–25% of cN0 cases harbour occult metastases, making it an overtreatment for most patients. To optimize selection, guidelines classify cN0 patients into low-, intermediate-, and high-risk groups based on pT stage, histological grade, and lymphovascular invasion [5, 11, 12].

Table 2 summarizes current recommendations on LN management**.** Surveillance is reported for low-risk patients across all guidelines (III, A), with EAU-ASCO extending it to intermediate-risk cases (T1a G2) (SE: weak). For intermediate- and high-risk tumors, guidelines agree on recommending dynamic sentinel node biopsy (DSNB) or radical or modified ILND, depending on availability and institutional expertise (III, A). DSNB, which targets the first draining lymph node(s), offers the lowest complication rates and high diagnostic accuracy for cN0 PC, particularly in high-volume centers. Its effectiveness improves when combined with ultrasound and fine-needle aspiration **(**FNA) cytology for targeted sampling of suspicious nodes**.** As it removes fewer nodes, DSNB is superior in reducing the risk of lymphoceles and other lymphatic complications (Table 2).

**Table 2:** Summary of methodological aspects and recommendations of the EAU-ASCO, ESMO-EURACAN, and NCCN guidelines on LN management in PC

Node stage and risk	ESMO-EURECAN	NCCN	EAU-ASCO
cN evaluation	Palpability	Palpability, number, unilateral/bilateral, dimensions, ± mobility MRI if difficult to assess	Palpability, number, unilateral/bilateral, dimensions, ± mobility
cN0 classification criteria	pT stage, grade, LVI	pT stage, grade, LVI, PNI	pT stage, grade, LVI, PNI
Low risk cN0 management	*Surveillance* (pTa/pTis and pT1G1) (III, A)	*Surveillance (PeIN, Ta, T1a)*	Surveillance (pTa, pTis and negative LVI pT1)
Intermediate risk cN0 management	*DSNB* (pT1G2) If not available *Modified ILND* ± *frozen section* (III, A)	modified or radical ILND or DSNB (T1b, G1–2)	surveillance vs DSNB/ILND (T1a G2 disease) (weak)
High risk cN0 management	*DSNB* (pT1-4G3/G4 or LVI+) (III, A) If not available *Modified ILND* ± *frozen section* (III, A)	Modified or radical ILND or DSNB (T1b, G3–4; T2 or greater)	DSNB/ILND (T1b or higher) (strong)
cN1 management	*Modified ILND*	FNA, with or without excisional biopsy Risk features (RF): T1, G3, LVI+; PNI+; low differentiation (> 50%) RF+: ILVD RF−: Percutaneous lymph node biopsy Biopsy-: Bilateral ILVD Neoadjuvant chemotherapy + ILVD Biopsy+: Strict surveillance Excisional biopsy	FNA, with or without excisional biopsy + 18FDG-PET/CT or CT (SE: strong) Open ILND (LE 2A; SE strong) fsILND (LE 2B)

*EAU-ASCO* European Association of Urology-American Society of Clinical Oncology, *ESMO-EURACAN* European Society for Medical Oncology-European Reference Network on Rare Adult Solid Cancers, *NCCN* National Comprehensive Cancer Network, *LN* lymph node, *PC* penile cancer, *LoE* level of evidence, *GRADE* Grading of Recommendations, Assessment, Development, and Evaluations, *cN* clinical nodal status, *MRI* magnetic resonance imaging, *LVI* lymphovascular invasion, *PNI* perineural invasion, *DSNB* dynamic sentinel node biopsy, *ILND* inguinal lymph node dissection, *ILVD* inguinal lymphadenectomy

### Clinically evident disease (cN1–2)

In patients with palpable LNs, nodal metastasis is observed in approximately 45–80% of cases [14]. Open inguinal lymph node dissection (ILND), as outlined by Daseler et al., continues to be the standard approach for cN1–2 disease (LE: 2a; SE: strong) (III, A) [15]. However, its extensive nature, which includes fascia lata removal, femoral vessel skeletonization, and sartorius muscle transposition, is associated with a considerable risk of complications (21–55%) [12]. To reduce morbidity, EAU-ASCO recommends fascial-sparing ILND (fsILND), which yields comparable oncological results in cN1 patients with fewer complications (29.3%) (LE: 2b, SE: strong). Noteworthy, this recommendation is based on results from a single retrospective study on 201 fsILND for patients with < N2 disease, demonstrating similar oncological outcomes to radical ILND, with a 3-year median disease-free survival (DFS) rate of 92.1% (100%, 91.3%, 80% and 33.3% for pN0, pN1, pN2, pN3 disease, respectively) [16]. Similarly, ESMO supports fsILND for cN1–2 cases when feasible (III, A) [17].

The NCCN guidelines emphasize that it is not possible to predict the laterality of inguinal nodal metastasis based on the location of the penile tumour in cN0 cases. Likewise, in patients with a unilateral palpable node, approximately 30% will have contralateral not palpable positive nodes [18]. Therefore, bilateral lymphadenectomy is recommended for patients undergoing ILND [19]. Furthermore, NCCN agrees on adopting ILND as the standard of care for cN1-N2 but emphasizes that in patients with bulky inguinal disease (> 3 cm), neoadjuvant chemotherapy prior to surgery may improve prognosis. In this setting, ILND is generally performed within 4–6 weeks after chemotherapy to reduce the risk of progression [20–22].

#### Minimally invasive ILND

Overall, guidelines consistently recognize the potential advantages of VEIL/RA-VEIL, including lower wound complications, shorter hospital stays, and comparable LN yields, but stress the need for further validation [23–25]. While RA-VEIL may reduce wound morbidity, there is no strong evidence supporting benefits in preventing post-operative lymphocele or lymphoedema rates, as the same number of nodes is removed [26–33]. Additionally, oncologic safety remains uncertain, since most studies focus on cN0 patients, indicating selection bias. Only a few studies have assessed its use in confirmed LN-positive disease, making it premature to establish oncological safety in this setting (LE: 2b). In this context, EAU explicitly recommends offering minimally invasive ILND to cN1–2 patients only within a clinical trial setting (SE: strong), while NCCN/ESMO refrain from making recommendations due to insufficient supporting evidence [34]. Finally, when surgical staging is indicated and DSNB is not available or if preferred by the patient VEIL/RA-VEIL may be offered (SE: strong).

### Clinically evident disease (cN3)

The management of PC at stage N3, characterized by clinically fixed and enlarged inguinal LNs (cN3) or clinically evident pelvic LN metastases, is approached through multimodal strategies as outlined in the EAU, ESMO, and NCCN guidelines. While specific recommendations vary, there is broad consensus regarding the importance of neoadjuvant chemotherapy (NAC) followed by radical surgery in patients who exhibit a positive response. In this context, EAU guidelines strongly recommend cisplatin- and taxane-based neoadjuvant chemotherapy (LE: 2b; SE: Strong), as emerged from a systematic review reporting a radiological response rate of 53% and a complete pathological response in 12.8% of patients [22, 35]. Radical inguinal and pelvic LN dissection (PLND) is recommended for patients who respond to NAC or do not show disease progression. The initiation of treatment with surgery in patients with cN3 is not recommended (SE: Weak), and minimally invasive techniques (robotic or laparoscopic) are considered inappropriate in this setting (SE: Strong) [36, 37].

NCCN also recommends cisplatin-based chemotherapy prior to surgery but specifies that inguinal and/or pelvic lymphadenectomy may be performed without NAC in patients who are ineligible for neoadjuvant chemotherapy. Both the EAU and NCCN consider chemoradiotherapy as a potential option for patients who are not candidates for standard chemotherapy, although the available evidence is limited (SE: Weak). EAU extends this recommendation to include adjuvant radiotherapy (with or without chemotherapy sensitization) for patients with pN2/N3 disease, including those who have previously received neoadjuvant chemotherapy or those who are not candidates for surgery. Conversely, NCCN emphasizes the need to perform a LN biopsy prior to administering neoadjuvant chemoradiotherapy. Furthermore, NCCN recommends bilateral pelvic lymphadenectomy if four or more inguinal LNs are found to be positive. Post-PLND, adjuvant radiotherapy or chemoradiotherapy may be considered for patients with positive pelvic LNs.

Regarding the adjuvant use of chemotherapy alone, EAU acknowledges limited supporting data; however, it may be offered to patients with pN3 disease following LND if NAC was not previously administered, following a thorough evaluation of the risks and benefits (LE: 4) [5].

With the same aim, ESMO guidelines concurs with the EAU and NCCN ones, recommending neoadjuvant chemotherapy followed by ipsilateral inguinal and pelvic lymphadenectomy in responsive patients (III, B). However, ESMO notes that contralateral dissection is contingent on clinical and pathological evaluation. In patients who do not respond to NAC or who are ineligible for surgery, ESMO also recommends chemoradiotherapy or participation in clinical trials (III, C) [38, 39].

Although current guideline recommendations focus primarily on chemotherapy-based multimodality treatment, recent studies have explored innovative systemic agents. EGFR-targeted therapies such as dacomitinib (pan-HER) have demonstrated promising activity, with a 32% objective response rate in a phase II trial involving patients with advanced or metastatic penile squamous cell carcinoma [40]. Likewise, immunotherapy with anti-PD-1 agents (both as monotherapy and in combination with chemotherapy or anti-EGFR drugs) has yielded pathological complete response rates exceeding 60% in early phase II studies [41]. These results are preliminary and require validation in larger prospective trials. Radiotherapy also remains an important palliative option for symptomatic nodal metastases with treatment tailored to the patient’s clinical condition and therapeutic goals [42].

### Pelvic LN dissection (PLND)

EAU emphasizes that, among the various predictors of LN node involvement, the number of positive inguinal LNs (1–2 vs. ≥ 3 or more, without extracapsular extension) is associated with pelvic LN positivity in a percentage of patients ranging from 0 to 6.5% for the first group, and from 33 to 67% for the second group [43, 44]. Moreover, the presence of extracapsular spread has been found to be significantly associated with the positivity of ipsilateral pelvic LNs in four studies [45]. Additional predictors of pelvic LN involvement reported include: (i) strong p53 immunoreactivity, (ii) LN density > 30%, and (iii) the grade of the primary tumor [44].

A multicenter retrospective study compared the outcomes of bilateral prophylactic pelvic LN dissection (pPLND) in patients with N2 or N3 disease versus no intervention, reporting a 5-year overall survival advantage in the pPLND group (35% vs. 25%), though statistical significance was not reached (*p* > 0.05) [46]. In N2 patients, 3-year survival was significantly higher in the PLND group compared to the non-surgical group (83.3% vs. 50.2%, *p* = 0.03). However, this difference was not observed in N3 patients. Therefore, the EAU recommends ipsilateral prophylactic pelvic LN dissection, performed either with an open or minimally invasive technique, in patients with three or more positive inguinal LNs on one side on pathological examination or in the presence of extracapsular extension on pathological examination (SE: Weak). NCCN underscores that patients with a single positive inguinal LN have a risk of pelvic LN involvement inferior to 5%, as reported by the Netherlands Cancer Institute [47]. For this reason, NCCN recommends PLND, including resection of external iliac, internal iliac, and obturator LNs, in patients with three or more positive inguinal LNs.

According to the NCCN, PLND may be performed during the same procedure as ILND, if intraoperative frozen section reveals positivity in three or more inguinal lymph nodes, or at a later stage, depending on the pathological characteristics of the ILND sample [48]. Consensus among guidelines is not reached regarding unilateral or bilateral PLND: NCCN supports that the presence of four or more positive inguinal lymph nodes justifies the performance of bilateral PLND, whereas unilateral PLND is recommended if three or fewer positive inguinal lymph nodes are present [49]. Finally, ESMO highlights that ipsilateral pelvic lymph node metastases are more frequent in patients with two or more positive inguinal lymph nodes, in the presence of extracapsular extension, or when the metastasis is > 30 mm in diameter. For this reason, ESMO recommends unilateral PLND in this setting of patients.

## Practical considerations & conclusion

We acknowledge the substantial heterogeneity that persists across current EAU-ASCO, ESMO-EURACAN, and NCCN recommendations for the management of LN metastases in PC. Much of this inconsistency reflects differences in local expertise, resource availability, and institutional volume. Access to key staging and treatment modalities—such as ultrasound-guided FNA, DSNB, VEIL or RA-VEIL—remains uneven across centres, and peri-operative and oncological outcomes are strongly volume-dependent, with high-volume units consistently achieving superior results. These structural disparities, together with the limited evidence base underpinning several aspects of LN management, help explain the lack of uniformity across guidelines and the challenges in translating them into consistent clinical practice. Nevertheless, a set of key considerations emerges from the collective perspective of the international guidelines:

- Open ILND remains the standard treatment for LN metastatic PC, as surveillance or delayed LND may result in missed curative opportunities.
- If DSNB is unavailable or not the preferred choice of the patient, VEIL/RA-VEIL can serve as an alternative approach. Compared to open ILND, VEIL/RA-VEIL emerges as a viable alternative, offering equivalent diagnostic accuracy while potentially reducing wound-related complications.
- Current comparative data on oncological outcomes remain limited, primarily based on cN0 cases, underscoring the need for further studies in cN+ patients.
- Pelvic LN dissection (PLND) should be considered in patients with ≥ 3 positive inguinal nodes or extracapsular spread, as these are the strongest predictors of pelvic involvement.
- Clinically advanced nodal disease (cN3) benefits from neoadjuvant chemotherapy followed by aggressive surgical resection when feasible.

Further collaborative efforts and comparative trial are needed to refine guideline recommendations and clarify the oncological impact of ILND.

## Data Availability

No datasets were generated or analysed during the current study.
